# Statistical classifiers for diagnosing disease from immune repertoires: a case study using multiple sclerosis

**DOI:** 10.1186/s12859-017-1814-6

**Published:** 2017-09-07

**Authors:** Jared Ostmeyer, Scott Christley, William H. Rounds, Inimary Toby, Benjamin M. Greenberg, Nancy L. Monson, Lindsay G. Cowell

**Affiliations:** 1 Department of Clinical Sciences, UT Southwestern Medical Center, 5323 Harry Hines Boulevard, Dallas, TX 75390-9066 USA; 2 Department of Neurology and Neurotherapeutics, UT Southwestern Medical Center, 5323 Harry Hines Boulevard, Dallas, TX 75390-9036 USA

**Keywords:** Antibody, Immune repertoire, CDR3, Machine learning, Multiple sclerosis, Statistical classifier

## Abstract

**Background:**

Deep sequencing of lymphocyte receptor repertoires has made it possible to comprehensively profile the clonal composition of lymphocyte populations. This opens the door for novel approaches to diagnose and prognosticate diseases with a driving immune component by identifying repertoire sequence patterns associated with clinical phenotypes. Indeed, recent studies support the feasibility of this, demonstrating an association between repertoire-level summary statistics (e.g., diversity) and patient outcomes for several diseases. In our own prior work, we have shown that six codons in VH4-containing genes in B cells from the cerebrospinal fluid of patients with relapsing remitting multiple sclerosis (RRMS) have higher replacement mutation frequencies than observed in healthy controls or patients with other neurological diseases. However, prior methods to date have been limited to focusing on repertoire-level summary statistics, ignoring the vast amounts of information in the millions of individual immune receptors comprising a repertoire. We have developed a novel method that addresses this limitation by using innovative approaches for accommodating the extraordinary sequence diversity of immune receptors and widely used machine learning approaches. We applied our method to RRMS, an autoimmune disease that is notoriously difficult to diagnose.

**Results:**

We use the biochemical features encoded by the complementarity determining region 3 of each B cell receptor heavy chain in every patient repertoire as input to a detector function, which is fit to give the correct diagnosis for each patient using maximum likelihood optimization methods. The resulting statistical classifier assigns patients to one of two diagnosis categories, RRMS or other neurological disease, with 87% accuracy by leave-one-out cross-validation on training data (*N* = 23) and 72% accuracy on unused data from a separate study (*N* = 102).

**Conclusions:**

Our method is the first to apply statistical learning to immune repertoires to aid disease diagnosis, learning repertoire-level labels from the set of individual immune repertoire sequences. This method produced a repertoire-based statistical classifier for diagnosing RRMS that provides a high degree of diagnostic capability, rivaling the accuracy of diagnosis by a clinical expert. Additionally, this method points to a diagnostic biochemical motif in the antibodies of RRMS patients, which may offer insight into the disease process.

**Electronic supplementary material:**

The online version of this article (10.1186/s12859-017-1814-6) contains supplementary material, which is available to authorized users.

## Background

Lymphocytes express immune receptors on their cell surface, the genes of which are somatically generated in developing lymphocytes through a DNA recombination process known as V(D)J recombination. V(D)J recombination assembles variable (V), diversity (D), and joining (J) gene segments into mature, composite genes. The diversity of gene sequences generated by V(D)J recombination is huge as a result of varying combinations of V, D, and J gene segments, as well as sequence modifications (e.g.*,* exonucleolytic activity and non-templated nucleotide addition) at the junctions of rearranged gene segments. As a result, each individual has millions of unique immune receptor genes. Somatic generation of a tremendously diverse repertoire of immune receptors enables effective immune responses against an essentially infinite array of antigens, such as those derived from pathogens or tumors, but it can also lead to detrimental effects, such as autoimmune responses and organ rejection following transplantation. The composition of immune repertoires shifts in response to such immunological events, and thus reflects previous and ongoing immune responses.

Deep sequencing of immune repertoires has made it possible to comprehensively profile the clonal composition of lymphocyte populations, opening the door for novel approaches to diagnose and prognosticate diseases with a driving immune component by identifying repertoire sequence patterns associated with important clinical phenotypes. Recent studies support the feasibility of this approach. Patterns in the relative abundances of V gene segment types in a repertoire have been observed in association with various autoimmune diseases [1–3], as well as with metastasis-free/progression-free survival in basal-like and HER2-enriched breast cancer subtypes and the immunoreactive ovarian cancer subtype [4]. Repertoire diversity has been associated with prognosis in gastric cancer [5] and with outcome following Ipilimumab treatment for metastatic melanoma [6]. We have demonstrated that VH4-containing genes in B cell repertoires from the cerebrospinal fluid of RRMS patients have higher replacement mutation frequencies at six codons than those in healthy controls [2, 7]. The sum of Z scores across the six codons can distinguish RRMS patients from those with other neurological diseases (OND) [7].

The methods applied to date for associating repertoire patterns with clinical phenotypes have focused on repertoire-level features, ignoring the vast amounts of information available in the millions of individual immune receptors comprising a repertoire. This has been due to difficulties accounting for the tremendous diversity of immune repertoires and the lack of methods for mapping the large number of individual sequences in a repertoire to a single phenotype label. We have developed a novel method that addresses both limitations by combining widely used machine learning methods with innovative approaches for accommodating the extraordinary sequence diversity of immune receptors and for aggregating the set of predictions made for each sequence in a repertoire.

We applied our method to RRMS, a subtype of multiple sclerosis (MS). MS is an autoimmune disease that is notoriously difficult to diagnose. It is believed to be the result of immune cells attacking the myelin insulation around nerve cells, leaving patients with physical and cognitive impairments. Unfortunately, there are no symptoms, physical findings, or lab tests that provide a definitive MS diagnosis. Patients have to demonstrate findings consistent with MS and simultaneously have alternative diagnoses be excluded [8]. Thus, reaching an MS diagnosis can be a slow process, but early detection is needed, because prompt intervention can significantly slow the progression of the disease [9].

We applied our method to B cell receptor (BCR) heavy chain genes to develop a statistical classifier that assigns patients to one of two diagnosis categories, RRMS or OND, based on the BCR heavy chain biochemical features. The classifier has 87% accuracy by leave-one-out cross-validation on training data (*N* = 23) and 73% accuracy on unused data from a separate study (*N* = 102). These results demonstrate the utility of our new method for identifying repertoire-based signatures with diagnostic potential.

## Results

Our overall approach was as follows. We used two data sets, one as training data and one as validation data (Table 1). The training data set was used with exhaustive leave-one-out cross-validation for model selection to identify the best model from among seven models tested (Table 2). The seven models correspond to different approaches to representing immune receptor sequences. The model with highest classification accuracy by cross-validation was selected for application to the validation data set.

**Table 1:** Repertoire sequencing data sets used to develop and test the MS classifier. The number of patients in each study with each diagnosis is shown

	Relapsing Remitting Multiple Sclerosis	Other Neurological Disease
2015 Study [7]	11	12
2017 Study	60	42

**Table 2:** Sequence Representations used for Model Selection. CDR3 sequences were cut into snippets of varying length and represented as DNA sequence, amino acid sequence, or Atchley factors [10]. Classification accuracy results are reported as the fraction of patients for which the model’s prediction of the diagnosis is correct

Snippet Length	Sequence Representation	Classification Accuracy on the Training Data Set by Exhaustive 1-Holdout Cross-Validation
4 Amino Acids	Atchley Factors	11/23 ≈ 47.8%
5 Amino Acids	Atchley Factors	15/23 ≈ 65.2%
6 Amino Acids	Atchley Factors	20/23 ≈ 87.0%
7 Amino Acids	Atchley Factors	14/23 ≈ 60.9%
2 DNA Triplets	DNA Nucleotides	12/23 ≈ 52.2%
6 DNA Triplets	DNA Nucleotides	8/23 ≈ 34.8%
6 Amino Acids	Amino Acid Residue	15/23 ≈ 65.2%

**Fig. 1:**
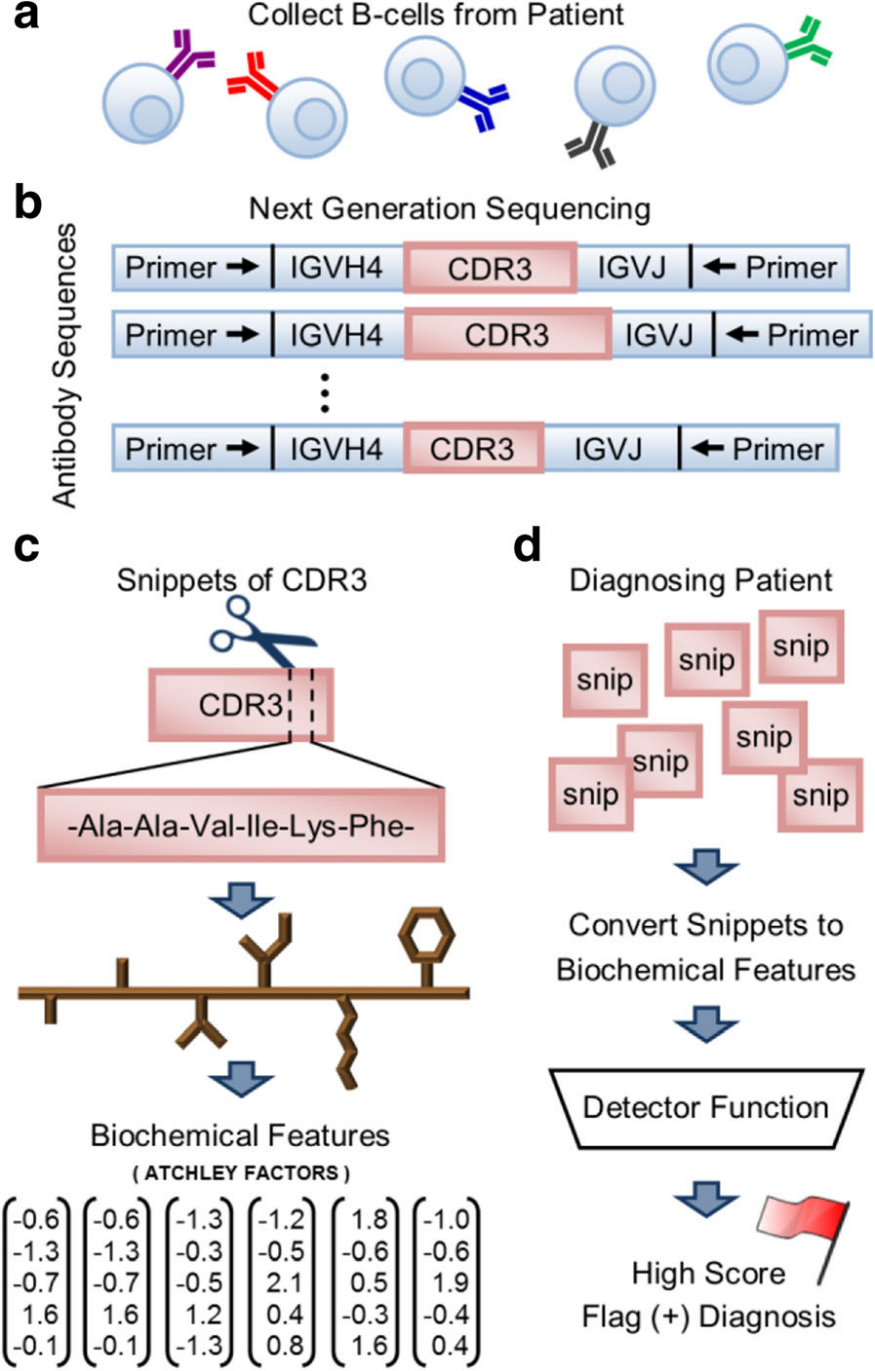
Study Overview (**a**) B cells are collected from patient cerebrospinal fluid. (**b**) DNA is extracted, and next generation sequencing is used to sequence immunoglobulin heavy chain loci expressing IGHV4 rearrangements. (**c**) Snippets of amino acid sequence taken from the CDR3 are converted into a set of chemical features using Atchley factors. (**d**) The chemical features are scored by a detector function. The detector function used in this study is the same function used in logistic regression. A positive diagnosis (for RRMS) is flagged whenever a high scoring snippet is found. Values for the weights on each Atchley factor as well as the bias term are determined by maximizing the likelihood of obtaining the correct diagnoses on a training set of patients

The training data set consisted of 23 patients, 11 with RRMS and 12 with OND (2015 Study, Table 1). The validation data set consisted of 102 patients, 60 with RRMS and 42 with OND (2017 Study, Table 1). For both studies, B cell repertoires were collected and processed as described in [7]. Briefly, samples were collected from patient cerebrospinal fluid (CSF) (Fig. 1a), and VH4-containing BCR heavy chain genes were sequenced using next generation sequencing (Fig. 1b). VH4-containing heavy chains were targeted because previous studies found elevated VH4 expression in patients with RRMS [2, 7]. Sequence pre-processing was performed as described in Methods to identify complementarity determining region 3 (CDR3) sequences for input into our method.

### Representing immune receptor sequences for statistical classification

We utilized the CDR3 sequence of each heavy chain gene, because it is the somatically generated portion of the gene and the primary determinant of the antigen binding specificity encoded by the gene. To accommodate the varying length of CDR3, each CDR3 sequence was cut into snippets of equal length (i.e.*,* k-mers). We considered snippet lengths of 2, 4, 5, 6, and 7 amino acids or codons. For each CDR3, the full set of overlapping snippets was used. We considered three different sequence representations: DNA sequence, amino acid sequence, and a representation based on Atchley factors (Fig. 1c). There are five Atchley factors derived from a set of over 50 amino acid properties by dimensionality reduction to identify clusters of amino acid properties that co-vary [10]. The five Atchley factors correspond loosely to polarity, secondary structure, molecular volume, codon diversity, and electrostatic charge. For the Atchley factor representation, each amino acid in a snippet is represented by a vector of its five Atchley factor values. We conducted model selection over seven combinations of snippet length and sequence representation (Table 2).

### Scoring each sequence in a repertoire

Every snippet from every CDR3 sequence in a patient’s repertoire is scored by a detector function indicating if a snippet predicts RRMS. We use a logistic function because of its widespread use and simplicity, and because it models the outcome of a two-category process. The first step is to compute a biased, weighted sum of the snippet’s features, referred to as a logit.

$$ \mathrm{logit}={b}_0+{W}_1\cdot {f}_1+{W}_2\cdot {f}_2+\cdots +{W}_N\cdot {f}_N(1) $$

For the DNA and amino acid sequence representations, the values *f*_1_ through *f*_*N*_ represent the snippet residues. For the Atchley factor representation, the *f*_*i*_ represent the five Atchley factors from each residue in the snippet. For snippets of length six, *N* = 30. The bias term *b*_0_ along with the weights *W*_*i*_ are the parameters of the model and are fit by maximum likelihood using gradient descent optimization techniques as described below. The same weights *W*_*i*_ and bias term *b*_0_ are used for all snippets. Once the logit is computed, the value is passed through the sigmoid function to obtain a score between 0 and 1 (Additional file 1: Figure. S1).

$$ \mathrm{score}=\frac{1}{1+{e}^{-\mathrm{logit}}}(2) $$

### Aggregation of snippet scores to predict a diagnosis

A patient’s snippet scores need to be aggregated into a single value to form a diagnosis. Because only a small fraction of BCRs in a patient’s repertoire are expected to be disease related, it is necessary to capture a diagnosis even if only a few snippets have a high score. This is accomplished by assigning a positive diagnosis when even a single high scoring snippet is found (Fig. 1d). Assuming the output of the detector function represents a probability value between 0 and 1, the form of the model can be written as:

$$ P\left(\mathrm{positive}\  \mathrm{diagnosis}|{\mathrm{snip}}_1,{\mathrm{snip}}_2,{\mathrm{snip}}_3,\dots \right)=\mathrm{Maximum}\left({\mathrm{score}}_1,{\mathrm{score}}_2,{\mathrm{score}}_3,\dots \kern0.5em \right)(3) $$

A probability >0.5 indicates a positive diagnosis (RRMS), whereas a value <0.5 indicates an OND diagnosis.

### Parameter fitting by gradient descent

Specific values for the weights *W*_*i*_ and bias term *b*_0_ in the detector function are determined using the patient diagnoses. The values must be chosen to maximize the likelihood that each predicted diagnosis is correct. To search for the optimal values, gradient optimization techniques can be used. With these techniques, each parameter is iteratively adjusted along the gradient in a direction that maximizes the log-likelihood, which in turn maximizes the likelihood that each predicted diagnosis is correct. The initial value for the bias term *b*_0_ is 0, and initial values for the weights are drawn at random according to ${W}_i\sim \mathcal{N}\left(0,{N}_{\mathrm{features}}^{-1}\right)$. Because the Adam optimizer, a gradient descent based method, has been shown to work well on a wide range of optimization tasks, it is used here [11]. The Adam optimizer is run for 2500 iterations with a step size of 0.01. The default values for the other Adam optimizer settings are: *β*_1_ = 0.9, *β*_2_ = 0.999, *ϵ* = 10^−8^.

A limitation of using a gradient descent based method is there is no guarantee of finding the globally optimal solution. Although the chosen detector function constitutes a linear model, the scores from every snippet are aggregated together in a non-linear fashion. Multiple local minima could exist. To address this, 10^5^ runs of Adam optimization, each starting from different initial parameters ${W}_i\sim \mathcal{N}\left(0,{N}_{\mathrm{features}}^{-1}\right)$, are used, and the best fit solution over all runs is used to diagnose new patients.

### Development of the MS classifier– Model selection and validation

**Fig. 2:**
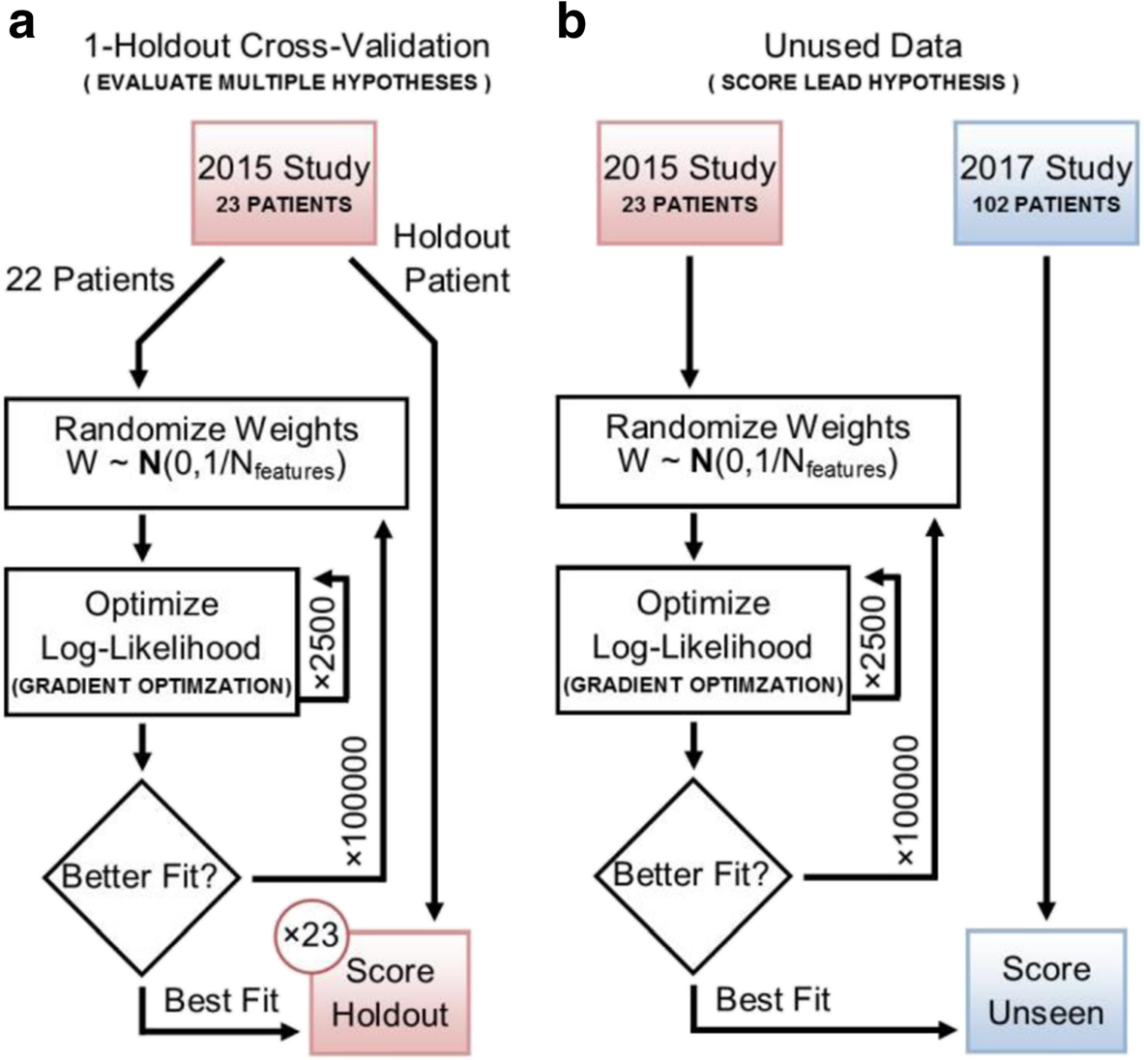
Workflow for Model Selection and Parameter Fitting. (**a**) The diagram shows how training data is used to train and evaluate multiple hypotheses. The model that gives the best classification accuracy on the exhaustive 1-holdout cross-validation constitutes the lead hypothesis. (**b**) The diagram shows how data is used to train and test the lead hypothesis. The best performing model is refitted to all the samples in the training data, and then used to score samples from the validation data set

**Fig. 3:**
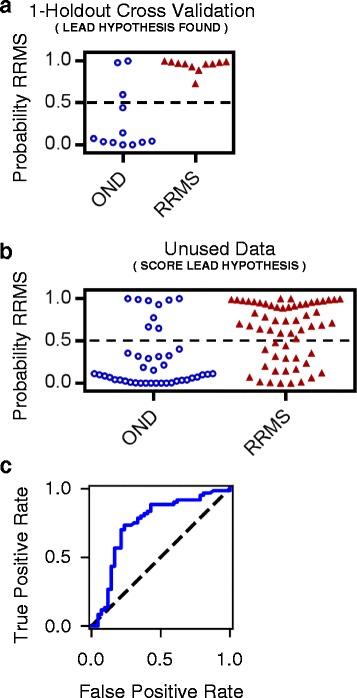
Classification Accuracy and Receiver Operating Characteristic (ROC) Curve. (**a**) Classification accuracy for the best performing model obtained via exhaustive 1-holdout cross-validation on training data. 87% of patients were correctly classified. (**b**) Classification accuracy of the best performing model on the validation data. 72% of patients were correctly classified. (**c**) The corresponding ROC curve shows true and false positive rates for different thresholds of a positive diagnosis based on the highest snippet score. The area under the curve is 0.75

We applied the above-described approach to our training data set of 23 patients using one-holdout cross-validation (Fig. 2a). Classification accuracy on the holdout patients was used to identify the best performing model from among the seven models tried (Table 2). A snippet size of 6 amino acid residues resulted in the highest classification accuracy. Categorical representations of the DNA nucleotides and amino acid residues both underperformed the Atchley factor representation. The best performing model correctly diagnosed 20 out of 23 patients (Table 2, Fig. 3a).

To estimate the probability of correctly classifying 20 of 23 patients by chance using our best performing model, we performed a permutation analysis. We performed 20 permutations in which patient diagnoses were permutated, and the one-holdout cross-validation procedure was conducted. The resulting classification accuracies are shown in Table 3. All were lower than 20 out of 23, allowing us to assign *p* < 0.05 to the observed accuracy. The average accuracy over all permutations is 40.4%.

**Table 3:** Classification accuracies observed by 1-holdout cross-validation after permuting diagnoses in our training data set. Results reported as the fraction of samples for which the model’s prediction of the diagnosis matches the label assigned under permutation

Classification Accuracy on the Training Data Set by Exhaustive 1-Holdout Cross-Validation (Labels Assigned under Permutation)				
7/23 ≈ 30.4%	11/23 ≈ 47.8%	6/23 ≈ 26.1%	6/23 ≈ 26.1%	7/23 ≈ 30.4%
15/23 ≈ 65.2%	13/23 ≈ 56.5%	11/23 ≈ 47.8%	12/23 ≈ 52.2%	7/23 ≈ 30.4%
18/23 ≈ 78.3%	7/23 ≈ 30.4%	6/23 ≈ 26.1%	8/23 ≈ 34.8%	11/23 ≈ 47.8%
4/23 ≈ 17.4%	11/23 ≈ 47.8%	9/23 ≈ 39.1%	10/23 ≈ 43.5%	7/23 ≈ 30.4%
Average: 40.4%				

To determine if the best performing model generalizes to unseen data, the full 23-patient training set was used to fit the weights and bias term, and the resulting model was applied to score a validation data set of 102 patients (Fig. 2b). The model correctly diagnoses 73 out of 102 patients, corresponding to an accuracy of 72% (Fig. 3b). The ROC curve for the validation data is shown in Fig. 3c. The area under the curve is 0.75.

### Analysis of the diagnostic biochemical motif

**Fig. 4:**
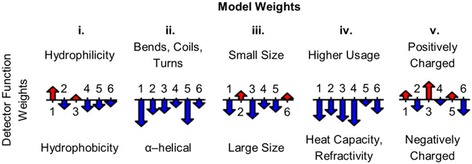
Illustration of the Classifier Weights. For each of the five Atchley factors, the weights for the model fit on all 23 training samples are shown for the six residue positions. Positive weight values are shown in *red* pointing up, and negative weight values are shown in *blue* pointing down. The length of the *arrow* corresponds to the weight’s magnitude

To discern the biochemical features of snippets resulting in a positive diagnosis, we examined the weights of the best performing model with parameters fit on the full 23-patient training set. The weights reveal how each Atchley factor contributes to the score and the relative importance of each position (Fig. 4). We observe relatively large, negative weights along almost every position of the snippet for Atchley factors II and IV, indicating a high probability of an RRMS diagnosis for snippets with negative values for these two Atchley factors. In particular, we notice large negative weights for factor II for positions 1 and 5 and for factor IV for positions 1, 3, and 4. A negative value for Atchley factor II correlates with amino acid residues that appear frequently in α-helical segments. A negative value for Atchley factor IV correlates with amino acid residues less commonly used and having high heat capacity and refractivity. The weights for the other Atchley factors are position-dependent. We observe relatively large positive weights for Atchley factor I at position 1 and for Atchley factor V at position 3. We also observe relatively large negative weights for positions 1 and 3 for Atchley factor III. This indicates increased probability of an RRMS diagnosis for snippets with large, positively charged, hydrophilic residues at snippet positions 1 and 3.

**Fig. 5:**
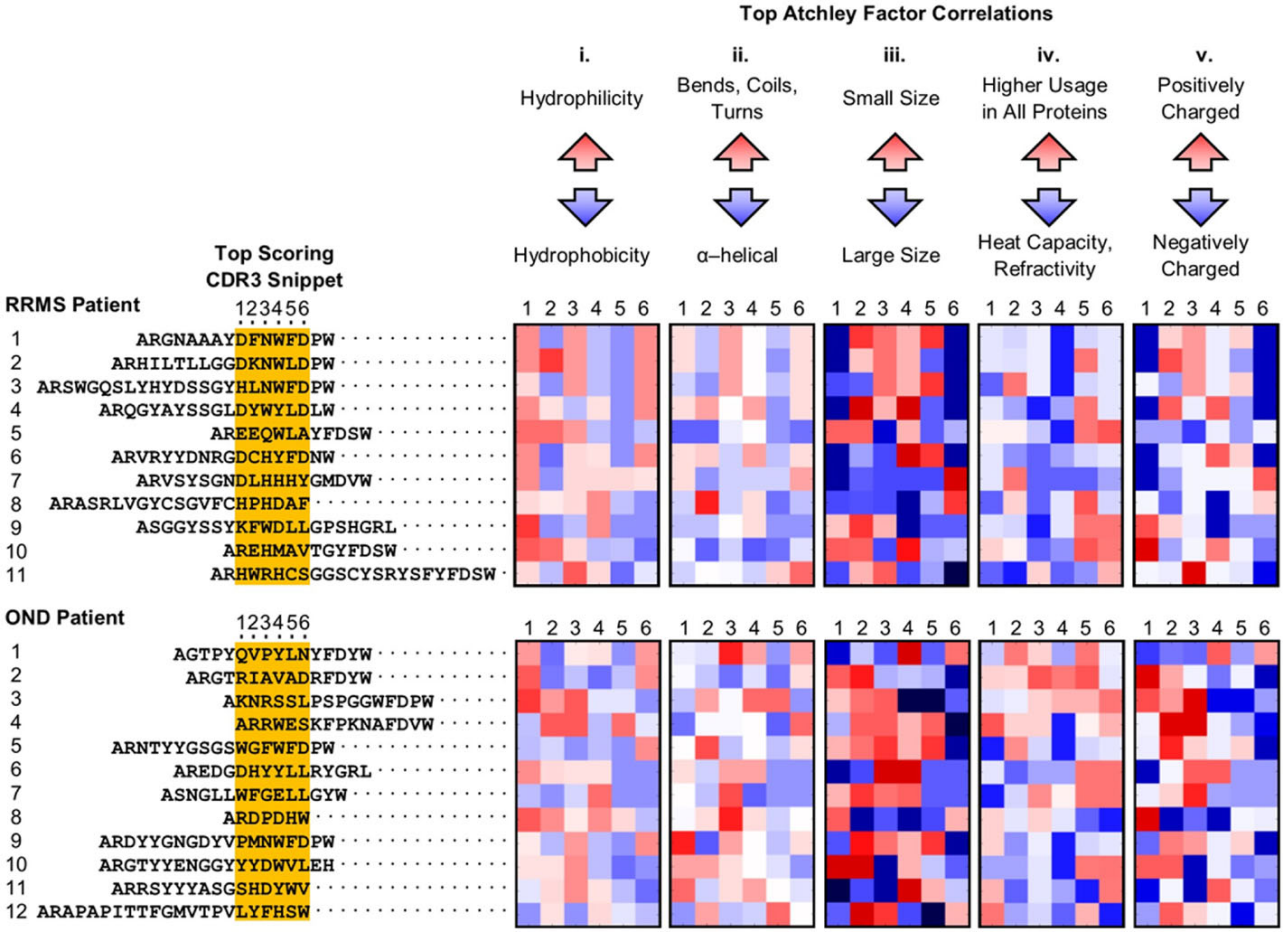
The Highest Scoring Snippet from each Patient in the Training Data Set. The snippets were scored with the model trained on all 23 subjects. (*Left*) Location of the snippet is shown in its CDR3 sequence using *yellow highlighting*. (Right) The Atchley factor values are shown for each snippet in the five *boxes*. Each *box* corresponds to one Atchley factor. The columns in each *box* correspond to the snippet positions

We next aligned the highest scoring snippet from each patient to determine where within CDR3 the diagnostic snippet is positioned (Fig. 5). We find that the highest scoring snippets can be located anywhere along CDR3. Although the snippet sequences do not align well, patterns are observable in their Atchley factors, which are shown next to each snippet (Fig. 5). Consistent with the values for the weights, we observe a tendency toward hydrophilicty for snippet position 1, toward α-helical values at position 5, toward high heat capacity and refractivity at positions 1 through 4, and toward negative charge at position 6.

**Fig. 6:**
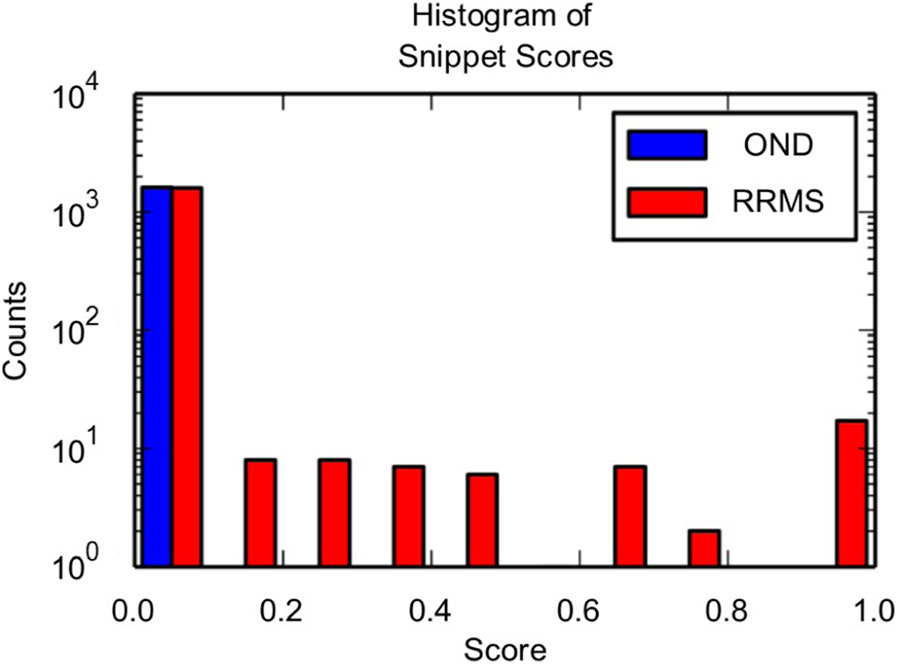
Histograms of Snippet Scores for all Snippets in the Training Data Set. The snippets were scored with the model trained on all 23 subjects. The *red bars* indicate the distribution of snippet scores from RRMS patients. The blue bars indicate the distribution of snippet scores from OND patients. Only a few snippets score above 0.5, which is diagnostic of RRMS

We next looked at the distribution of snippet scores in the 23 patients of our training data set (Fig. 6). Only 27 of 3259 snippets score above 0.5 (the threshold for a RRMS diagnosis), and all of these were from RRMS patient repertoires. Each RRMS patient had no more than 5 snippets that scored above the threshold.

**Fig. 7:**
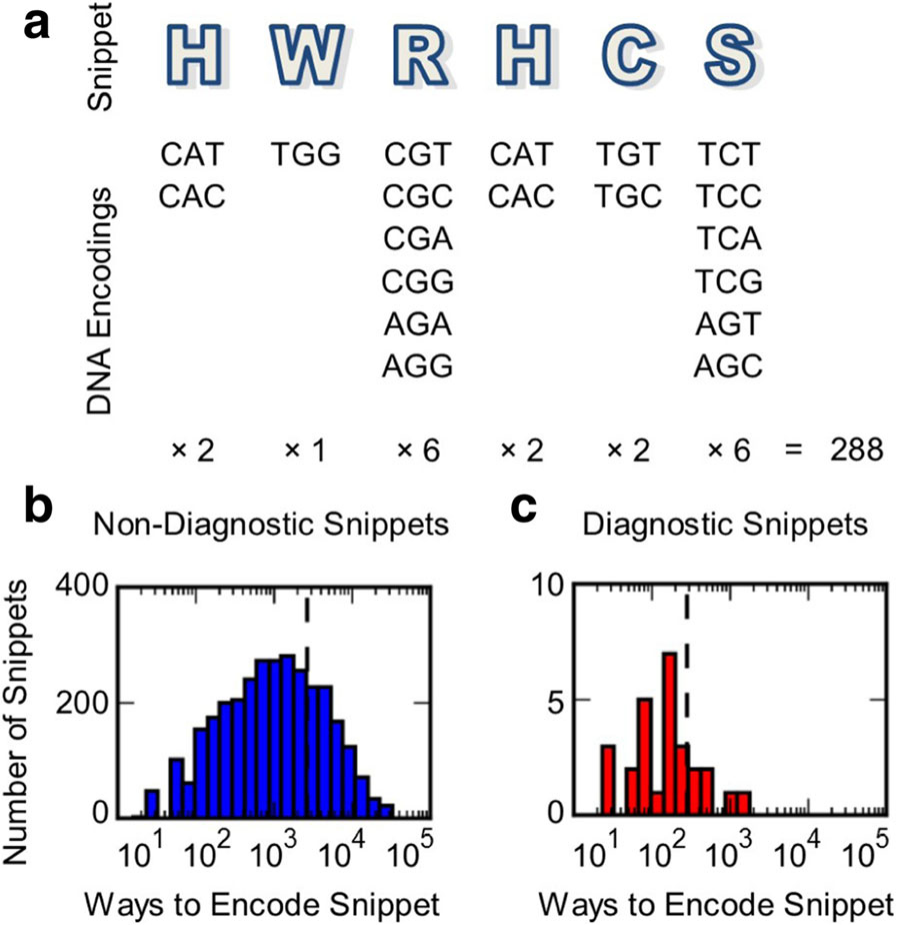
Encoding Degeneracy of Diagnostic and Non-diagnostic Snippets in the Training Data Set. (**a**) An example of how to calculate the number of ways a snippet can be encoded. (**b**) The distribution of the number of encodings for each non-diagnostic snippet. (**c**) The distribution of the number of encodings for each diagnostic snippet

To determine if the rarity of high scoring snippets in our patient repertoires can be attributed to the likelihood of the corresponding DNA sequences arising by chance in V(D)J recombination junctions, we examined the DNA encodings of each snippet. For each amino acid sequence, there are many possible DNA encodings. An example of how to calculate the total number of encodings for a single snippet is shown in Fig. 7a. The distribution for the total number of ways to encode each snippet is shown for non-diagnostic snippets in Fig. 7b and for diagnostic snippets in Fig. 7c. We find that the diagnostic snippets identified by the model have significantly fewer possible encodings than non-diagnostic snippets (*p*-value is 7.41 × 10^−8^). Under the naïve assumption that CDR3 sequence is generated at random, RRMS diagnostic snippets would be some of the least likely to occur.

## Discussion

High-throughput sequencing of immune repertoires now enables their detailed characterization, driving interest in utilizing repertoires in clinical applications, including diagnosing and prognosticating diseases (e.g.*,* [12]). Attempts to date have taken the approach of computing repertoire-level summary statistics, such as gene segment usage statistics, repertoire diversity, and clonality, and looking for differences in these statistics between two sets of repertoires (e.g.*,* cases and controls) [1–7]. This approach captures important features of a repertoire as a whole, and it can give insight into the biological processes underlying repertoire differences, such as whether there is clonal expansion or recruitment of new cells. On the other hand, this approach ignores the vast amount of information available in the individual immune receptor sequences, in particular, information about the encoded antigen binding specificities.

We present here a new approach that allows application of standard machine learning techniques to mine the full set of repertoire sequences for sequence patterns that distinguish one group of repertoires from the other. There are two key features of our approach. The first is that we capture all k-mers from all CDR3s in a repertoire and represent them as biochemical features using Atchley factors. The second is that we score all k-mers in a repertoire and then aggregate the set of scores to predict a label for the whole repertoire.

We have focused on the CDR3 portion of immune receptor sequences, because it is the somatically generated portion of the gene and the primary determinant of the antigen binding specificity encoded by the gene. The approach could be readily applied, however, to other parts of the gene, and even to the full sequence. The longer the sequence used, however, the more training data would be required to accommodate the corresponding increase in the number of model parameters.

To accommodate variation in CDR3 length, we represent each CDR3 sequence as a set of overlapping k-mers of a specified length. For example, a CDR3 of eight amino acids in length would be represented as three 6-mers. In our MS application, we used k-mers varying from four to seven amino acids and found that the highest classification accuracy was achieved with 6-mers (Table 2). Some CDR3s in our data sets are only six amino acids in length and are thus excluded from analysis when longer k-mers are used. Shorter k-mers, on the other hand, are more likely to appear in both MS and OND repertoires and are therefore not useful for discrimination.

We hypothesized that using a biochemical representation of amino acid k-mers would be beneficial, because such a representation captures sequence features related to receptor-antigen binding, and therefore related to the receptor’s function. Additionally, immune receptors with distinct amino acid sequences that bind to the same antigen would be expected to have similar biochemical properties. Indeed, we found that, for a fixed k-mer length of six amino acids, the biochemical representation resulted in higher classification accuracy than either an amino acid or DNA sequence representation (Table 2).

To aggregate the scores from all k-mers in a repertoire to a repertoire-level label, we took the maximum score based on the assumption that, among all receptors in a repertoire, those participating in the phenotype-related immune response may be rare. Thus, we wanted a function that would flag a positive diagnosis even for a single high-scoring snippet. The maximum score is a special case of the generalized mean, however, and other means, or even other functions, could be used to accommodate different assumptions about the underlying immune response and its role in the phenotype.

Using this approach, we were able to mine the individual CDR3 sequences of OND and RRMS patient repertoires to discover a biochemical motif that correctly classifies repertoires according to diagnosis with accuracy of 87% on training data and 72% on validation data. Importantly, no prior knowledge of the disease was utilized (i.e.*,* it was not necessary to know which antigens the B cells may be responding to). Additionally, the method did not rely on finding “public clones”, as we removed all shared sequences to control for possible carry-over contamination, as described in Methods.

In the context of MS, a classification accuracy of 72% is highly significant. MS is an autoimmune disease that is difficult to diagnose. There are no single symptoms, physical findings, or laboratory tests that provide a definitive MS diagnosis [13]. The current method of diagnosis relies on the 2010 revisions to the McDonald criteria and requires demonstration of dissemination of central nervous system lesions in both space and time, along with the exclusion of other diagnoses [8]. Currently, the most widely used piece of paraclinical evidence for MS diagnosis is magnetic resonance imaging (MRI). Therefore, the accuracy obtained using MRI to distinguish patients with MS from those with OND is the most appropriate direct comparison for our 72% accuracy. We know of one study based on the most recent MRI criteria making this assessment. An accuracy of 57% was observed for distinguishing MS from primary and secondary central nervous system vasculitis, lupus, and Sjogren’s syndrome [14]. In this context, a classification accuracy of 72% is highly significant.

Overfitting is a common concern with machine learning approaches and is usually addressed by regularization. To determine if the use of regularization could have improved our model’s performance on unseen data, we re-ran our procedure using early stopping, L1/L2 regularization, and bagging. The results are presented in supplementary materials (see Additional file 2). Only early stopping led to better performance. The classification accuracy by one-holdout cross-validation was 20 out of 23. The classification accuracy on the validation data set was 75 out of 102 (this is an improvement with two additional patients being correctly diagnosed). Because we had already un-blinded ourselves to our validation data set prior to applying these regularization techniques, we include these results only as supplementary material and not as the primary result reported in the paper.

The simple model presented here represents a first step in developing a new family of methods for applying machine learning to immune receptor sequences and is not without limitations. First, under the assumption that CDR3 sequences are generated at random during V(D)J recombination, and in the absence of negative selection against particular sequences, every possible snippet would be expected to appear in a sufficiently large repertoire. In this case, the mere presence of individual high-scoring snippets would not be sufficient to differentiate patients by their diagnosis, and more sophisticated aggregation functions would be needed. Second, our approach is designed to work for phenotypes for which the underlying adaptive immune response is driven by a restricted set of antigens that is common across patients. Thus, the approach may not be directly applicable to other immune response types, such as those against cancer neoantigens. Finally, while the approach is designed to be applicable to both BCR and T cell receptor (TCR) CDR3 sequences, the restriction on TCR to recognize antigen in the context of major histocompatibility complex molecules may limit the utility of our approach when applied to TCR. Our future work will include improvements to the method designed to address these issues.

## Conclusion

Immune repertoire sequencing is a promising new technology for studying adaptive immune responses. The point of this study is to lay the groundwork for statistical classifiers of repertoire sequence data. The work presented here represents a first in combining maximum likelihood with a statistical model that maps a set of immune receptor sequences to a single diagnosis. Other published methods require that the repertoire first be reduced to a fixed number of features. The model represents an initial step in developing a new family of methods for analyzing immune repertoires. Without relying on disease-specific knowledge about MS, a classifier has been built using novel statistical methods to correctly diagnose a significant fraction of patients with either RRMS or OND. The work presented here is further demonstration that diseases can be diagnosed from a sample of a patient’s immune repertoire. In the future, we expect to improve the diagnostic accuracy of our approach and to broaden its use to other diseases.

## Methods

The BCR heavy chain repertoires used in this study were collected in the context of other studies and shared with us upon request ([7] and under review with *Multiple Sclerosis Journal*). The samples from each study are referred to by the year the study was completed (Table 1). The repertoires were obtained as described in [7]. Briefly, CSF was taken by lumbar puncture from patients diagnosed with either RRMS or OND. DNA was extracted from CSF cell pellets, and targeted PCR was conducted to amplify rearranged BCR genes. Because of the limited amount of DNA extracted from each CSF sample, the PCR amplification protocol has been designed and carefully implemented to minimize the impact of amplification bias and carry-over contamination between samples. DNA is first amplified using whole genome amplification followed by targeted PCR amplification of the variable region of BCR heavy chain genes utilizing a V gene segment from the VH4 family. VH4 sequences were targeted because previous studies found VH4 usage to be elevated in patients with RRMS [15, 16]. Sequencing was conducted on the 454 platform. All tissue and patient data from both studies was handled in accordance with IRB-approved protocols.

The DNA sequences for each sample were processed to prepare them for analysis following recommendations in [17]. Specifically, sequences with a length less than 300 base pairs or an average quality score less than 35 were removed. The regions of each sequence to which the PCR primers hybridize were trimmed, and duplicate sequences appearing within a single sample were counted and then collapsed to a single sequence. The remaining sequences were aligned to a database of germline gene segments for V, D, and J gene assignment. Sequences representing non-functional rearrangements were removed. Processing was performed using the pRESTO [18], IgBlast [19], and RepCalc pipelines on the VDJServer Immune Repertoire Analysis Portal (http://www.vdjserver.org).

For the sequences remaining after processing, the CDR3 nucleotide sequences were identified, according to the Immunogenetics Information System (http://www.imgt.org) definitions. CDR3 sequences containing ambiguous base calls were removed, and the remaining sequences were compared across samples to identify potential carry-over contamination. The amount observed was in line with other studies [20, 21]. Sequences observed in more than one sample were removed. The remaining CDR3 sequences were used as input to develop the statistical classifier as described above.

Model development was implemented in TensorFlow, an open source machine learning package published by Google [22]. The data is represented as a 3-dimensional tensor of the form [Patient, Snippet, Atchley Factors]. The Atchley factors for each snippet are scored using TensorFlow functions for logistic modelling. The output is a tensor with a shape of [Patient, Score]. For each patient, the maximum score is used to represent their diagnosis. Taking the maximum score produces a new 1-dimensional tensor with a shape of [Probability]. Model parameters are fitted using gradient optimizers included with TensorFlow. Our project’s source code is available from https://github.com/jostmey/MaxSnippetModel​.

## Additional files

Additional file 1: Figure. S1. Plot of the logistic function used as part of the detector function to convert the logit value to a score between 0 and 1. (DOCX 50 kb)

Additional file 2: This document describes the results of applying four different approaches for mitigating overfitting. (DOCX 94 kb)

## Abbreviations

BCR: B cell receptor
CDR3: complementarity determining region 3
CSF: Cerebrospinal fluid
D: Diversity gene segment
HER2: Human epidermal growth factor receptor 2
IGHV4: heavy chain rearrangements utilizing a V gene segment from the VH4 family
IRB: Institutional review board
J: Joining gene segment
MRI: Magnetic resonance imaging
MS: Multiple Sclerosis
OND: Other neurological disease
PCR: Polymerase chain reaction
ROC: Receiver operating characteristic
RRMS: Relapsing Remitting Multiple Sclerosis
TCR: T cell receptor
V: Variable gene segment
VH4: Family 4 of V gene segments of the heavy chain locus
