# Supplementary figures and images for: Statistical classifiers for diagnosing disease from immune repertoires: a case study using multiple sclerosis
Source: BMC Bioinformatics. 2017 Sep 7;18:401. doi: 10.1186/s12859-017-1814-6 (PMC5588725; doi:10.1186/s12859-017-1814-6)

Figure S1


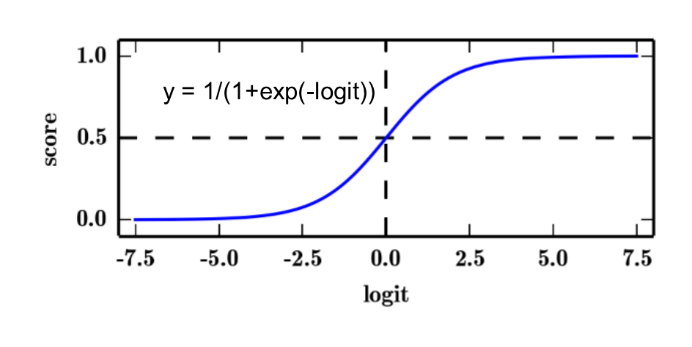

Supplement: Supplementary file 1 — Plot of the logistic function used as part of the detector function to convert the logit value to a score between 0 and 1. (DOCX 50 kb) [file 12859_2017_1814_MOESM1_ESM.docx]
