# Supplementary material for: Statistical classifiers for diagnosing disease from immune repertoires: a case study using multiple sclerosis
Source: BMC Bioinformatics. 2017 Sep 7;18:401. doi: 10.1186/s12859-017-1814-6 (PMC5588725; doi:10.1186/s12859-017-1814-6)
Supplement: Supplementary file 2 — This document describes the results of applying four different approaches for mitigating overfitting. (DOCX 94 kb) [file 12859_2017_1814_MOESM2_ESM.docx]

**Supplementary Material: Post-hoc Analysis of Overfitting**

*Whenever a statistical classifier is being fitted to a set of training data, overfitting is always a major concern. We performed a post-hoc analysis of our model to determine if other models could have been developed that would have generalized better to unseen data.*

***Effect on Overfitting of 100,000 Initial Starts***

With our approach, the initial weights are sampled from the distribution $W_{i}\mathcal{\sim N}(0,N_{\text{features}}^{-1})$ and the bias term is set to 0. Then, 2500 steps of ADAM iteration are used to fit the model. This fitting procedure is repeated many times to find the best fit to the training data (*i.e.*, to the 22 included patient samples). Each time the fitting procedure is repeated, the weight terms are initialized to a new set of values according to the distribution $W_{i}\mathcal{\sim N}(0,N_{\text{features}}^{-1})$ and the bias term is reset to 0. To address the reviewer’s concern that 100,000 sets of initial values is too large and leading to overfitting, we applied our approach with a varying number of starts ranging from 1 to 100,000. In each case, we assess how well the best fit model generalizes to the hold out data. The results are presented in the table below and show that as the number of starts increases, the best model generalizes better to unseen data, as measured by the classification accuracy and the average log likelihood on the holdout samples.

In each row, the snippet length is 6 amino acid residues and Atchley factors are used to represent the sequence.

| **Number of tries** | **Classification Accuracy on the Training Data Set by Exhaustive 1-Holdout Cross-Validation** | **Average Log-Likelihood on the Training Data Set by Exhaustive 1-Holdout Cross-Validation** |
| --- | --- | --- |
| 1 | 16/23 ≈ 69.6% | -1.257 bits |
| 10 | 17/23 ≈ 73.9% | -1.627 bits |
| 10^2^ | 16/23 ≈ 69.6% | -0.979 bits |
| 10^3^ | 19/23 ≈ 82.6% | -0.927 bits |
| 10^4^ | 19/23 ≈ 82.6% | -0.957 bits |
| 10^5^ | 20/23 ≈ 86.9% | -0.750 bits |

***Effect on Overfitting of 2,500 Iterations***

To determine if 2,500 steps of ADAM iterations is optimal, we performed early stopping. We performed 100,000 runs of gradient descent, running 10,000 steps of ADAM iterations for each one. At each step, we picked the best fit model from among all 100,000 models, as measured by the log-likelihood of the training data. We then computed the average log likelihood over the exhaustive 1-holdout cross validation at each step. In the figure below, the green trace shows the average log-likelihood fit to the training data and the red trace shows the average log-likelihood over the holdout samples.


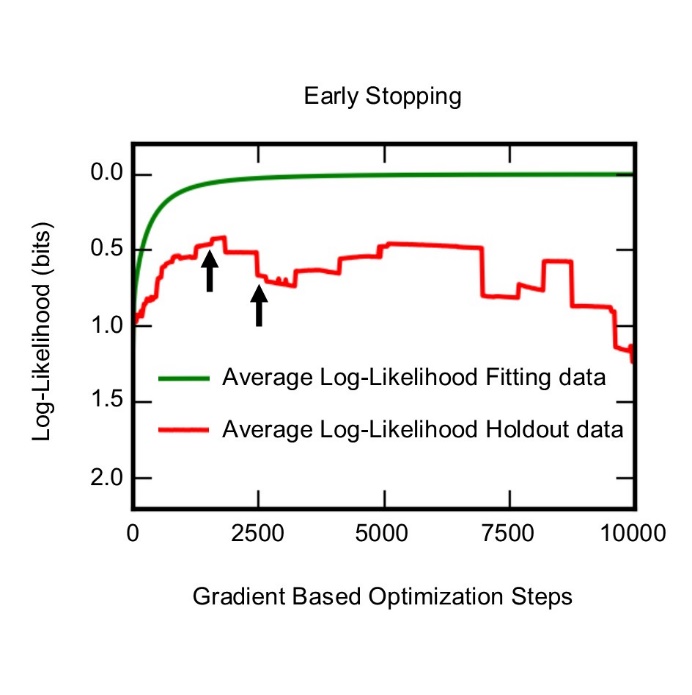


The analysis reveals that the model generalizes best to unseen data at approximately 1,300 steps of ADAM optimization.

We then fit the model using all 23 training samples (instead of performing a 1-holdout cross-validation) for 1,300 steps, picking the best fit out of 100,000 runs and applied the model to our validation set of 102 samples. The table below summarizes the results. We find that the classification accuracy on the validation data increases from 73/102 to 75/102, confirming improved generalizability from running fewer ADAM iterations.

| **Iterations of ADAM** | **Classification Accuracy on the Training Data Set by Exhaustive 1-Holdout Cross-Validation** | **Log-Likelihood on the Training Data Set by Exhaustive 1-Holdout Cross-Validation** | **Classification Accuracy of the Test Data from 2017 Study** |
| --- | --- | --- | --- |
| 1300 | 20/23 ≈ 86.9% | -0.475 bits | 75/102 ≈ 73.5% |
| 2500 | 20/23 ≈ 86.9% | -0.750 bits | 73/102 ≈ 71.6% |

***Effect of Regularization on Overfitting***

To determine if either L1 or L2 regularization might improve the performance of the model, we assessed the model’s performance across multiple L1 and L2 regularization constants. The results are presented in the table below. In each row, the snippet length is 6 amino acid residues and Atchley factors are used to represent the sequence. Our gradient descent based fitting procedure is run for 2,500 steps and the best fit to the seen data out of 100,000 runs is used to score the holdout sample. The classification accuracy and log-likelihood fit are then calculated over the unseen holdout data.

| **Regularization**  **Constant** | **Classification Accuracy on the Training Data Set by Exhaustive 1-Holdout Cross-Validation** | **Average Log-Likelihood on the Training Data Set by Exhaustive 1-Holdout Cross-Validation** |
| --- | --- | --- |
| No Regularization | 20/23 ≈ 86.9% | -0.750 bits |
| L1=10^-3^ | 20/23 ≈ 86.9% | -0.872 bits |
| L1=10^-4^ | 20/23 ≈ 86.9% | -0.872 bits |
| L1=10^-5^ | 20/23 ≈ 86.9% | -0.853 bits |
| L1=10^-6^ | 20/23 ≈ 86.9% | -0.846 bits |
| L2=10^-3^ | 13/23 ≈ 56.5% | -1.309 bits |
| L2=10^-4^ | 15/23 ≈ 65.2% | -1.456 bits |
| L2=10^-5^ | 20/23 ≈ 86.9% | -0.879 bits |
| L2=10^-6^ | 20/23 ≈ 86.9% | -0.881 bits |

In each case, adding the regularization penalty led to worse performance. The results are not surprising. With 30 weights and a bias term, there were only 31 parameters in the model, not much more than the 23 labeled samples being used.

***Effect of Bagging on Overfitting***

To see if bagging (a.k.a. bootstrap aggregation) can be used to alleviate overfitting, we proceeded as follows. From our training set of 23 patients, we removed the holdout sample. We then generated 100,000 bags of 22 patients each by sampling with replacement from the 22 non-holdout samples. For each bag, one attempt was made to fit the model to the data (instead of taking the best fit out of 100,000 tries). For each bag, 2500 steps of ADAM optimization were used. The initial weights were drawn from the distribution $W_{i}\mathcal{\sim N}(0,N_{\text{features}}^{-1})$, and the bias term was started at 0. Predictions were made from the many models by taking the label assigned by the majority of models (voting).

To apply bagging to the validation data set, we created 100,000 bags of 23 patients by sampling with replacement from our 23 training samples. We fit a model for each bag, and used each model to score the 102 patients. The predicted diagnosis for each patient was predicted by voting as described above.

| **Iterations of ADAM** | **Classification Accuracy on the Training Data Set by Exhaustive 1-Holdout Cross-Validation** | **Classification Accuracy of the Test Data from 2017 Study** |
| --- | --- | --- |
| 2500 | 17/23 ≈ 73.9% | 72/102 ≈ 70.6% |

The results show that the bagging procedure leads to worse performance on the exhaustive 1-holdout cross-validation. However, the model performed nearly as well on the test dataset.
